# Congenital Factor Deficiencies in Children: A Report of a Single-Center Experience

**DOI:** 10.1177/1076029617731596

**Published:** 2017-10-19

**Authors:** Zafer Şalcıoğlu, Cengiz Bayram, Hülya Şen, Gizem Ersoy, Gönül Aydoğan, Arzu Akçay, Deniz Tuğcu, Ferhan Akıcı, Müge Gökçe, Metin Demirkaya, Ali Ayçiçek, Zafer Başlar

**Affiliations:** 1 Department of Pediatric Hematology and Oncology, İstanbul Kanuni Sultan Süleyman Education and Research Hospital, İstanbul, Turkey; 2 Department of Hematology–Internal Medicine, Cerrahpaşa Medical School, İstanbul University, İstanbul, Turkey

**Keywords:** congenital factor deficiencies, prophylaxis, treatment

## Abstract

Congenital factor deficiencies (CFDs) refer to inherited deficiency of coagulation factors in the blood. A total of 481 patients with CFDs, who were diagnosed and followed at our Pediatric Hematology and Oncology Clinic between 1990 and 2015, were retrospectively evaluated. Of the 481 cases, 134 (27.8%) were hemophilia A, 38 (7.9%) were hemophilia B, 57 (11.8%) were von Willebrand disease (vWD), and 252 (52.3%) were rare bleeding disorders (RBDs). The median age of the patients at the time of diagnosis and at the time of the study was 4.1 years (range: 2 months to 20.4 years) and 13.4 years (range: 7 months to 31.3 years), respectively. The median duration of the follow-up time was 6.8 years (range: 2.5 months to 24.8 years). One hundred nineteen (47.2%) of 252 patients with RBDs were asymptomatic, 49 (41.1%) of whom diagnosed by family histories, 65 (54.6%) through preoperative laboratory studies, and 5 (4.2%) after prolonged bleeding during surgeries. Consanguinity rate for the RBDs was 47.2%. Prophylactic treatment was initiated in 80 patients, 58 of whom were hemophilia A, 7 were hemophilia B, 13 were RBDs, and 2 were vWD. Significant advances have been achieved during the past 2 decades in the treatment of patients with CFDs, particularly in patients with hemophilias. The rarity and clinical heterogeneity of RBDs lead to significant diagnostic challenges and improper management. In this regard, multinational collaborative efforts are needed with the hope that can improve the management of patients with RBDs.

## Introduction

Congenital factor deficiencies (CFDs) refer to a group of inherited bleeding disorders that results from the deficiency of coagulation factors in the blood. Rare encountered CFDs, known as “rare” bleeding disorders (RBDs), comprise deficiency of fibrinogen, factor (F) II, FV, combined FV and FVIII, FVII, FX, FXI, FXIII, and vitamin K-dependent factors, which are transmitted autosomal recessive with the exception of some cases of FXI deficiency and dysfibrinogenemia that may be autosomal dominant. The most common coagulation disorders, following von Willebrand disease (vWD), are hemophilia A and hemophilia B, which are X-linked transmitted and characterized by deficiency or absence of FVIII and FIX, respectively. Although hemophilias have similar prevalence around the world, it shows variations for RBDs.^1–4^ The incidence of RBDs is reported to be higher particularly in the countries where consanguineous or endogamous marriages are common, as in Turkey and other middle east countries, compared to Western populations.^1,5^ There are many guidelines and organizations for the proper diagnosis, management, and care of hemophilias.^6–15^ In contrast to hemophilias, knowledge of RBDs and their management are suboptimal since these conditions are rarely encountered and show variability in clinical presentations even within the same disorder, frequently with no family history to suggest an inherited bleeding disorder, leading to delayed or misdiagnosis and serious bleeding complications.^1,3^ Moreover, there is currently no comprehensive and worldwide epidemiologic data for RBDs that can facilitate timely and accurate diagnosis of these disorders.^1^ However, despite the challenges as mentioned above, there are several published clinical registries/databases and few guidelines with the aim to diagnose and manage the patients with RBDs.^16–23^ In this respect, it is very important to collect and share the data of the patients with CFDs, particularly of with RBDs, to establish a worldwide and comprehensive data for making accurate and timely diagnosis, thus contributing proper management and follow-up. In view of these knowledge, the present study aimed to evaluate the patients with CFDs, including vWD, hemophilias, and RBDs, who were diagnosed and followed up since 1990 at our hematology oncology clinic.

## Materials and Methods

A total of 481 patients who were diagnosed and followed up at İstanbul Kanuni Sultan Süleyman Training and Research Hospital, Department of Pediatric Hematology and Oncology, between 1990 and 2015, were included in the study. Clinical and laboratory data of patients were reviewed from patients’ files and electronic records that were available after 2005. Complete blood count, prothrombin time, partial thromboplastin time, plasma fibrinogen levels, bleeding time, and peripheral blood smears were performed by our hospital laboratory. Factor activity and inhibitor levels were tested by our hospital hemostasis laboratory and the hematology laboratory of İstanbul University, Cerrahpaşa Faculty of Medicine. Genetic analyses were performed in only 8 patients with FVII deficiency and 2 with hemophilia A, whereas it could not be performed for the other patients due to limited health-care resources. The classification recommended by the European Network of Rare Bleeding Disorders group was taken into consideration in the evaluation of clinical phenotypes of the patients with RBD.^23^ Patients were categorized into 4 groups according to clinical bleeding severity. In asymptomatic patients, there were no documented episodes of bleeding. Grade I bleeding was defined as bleeding that occurred after trauma or drug ingestion (antiplatelet or anticoagulant therapy). Grade II bleeding was defined as spontaneous minor bleeding (bruising, ecchymosis, minor wounds, oral cavity bleeding, epistaxis, and menorrhagia). Grade III bleeding was defined as spontaneous major bleeding (hematomas [intramuscular requiring hospitalization], hemarthrosis, central nervous system [CNS], gastrointestinal [GIS], and umbilical cord bleeding). Orthopedic surgeries and physiotherapy needs of the patients were discussed by our hospital’s hemophilia committee and then therapies were organized according to council decisions. Factor concentrates, fresh frozen plasma (FFP), or tranexamic acid as an antifibrinolytic agent were used for the treatment of bleedings, depending on the type of factor deficiency. Peripheral venipuncture was used for the administration of all clotting factors, and central venous access devices were not used. Patients’ demographic data including median age of patients at diagnosis and at the time of the study and the duration of follow-up time and prophylaxis were presented as median and range. Clinical and laboratory data were provided as number and percentage.

## Results

**Figure 1.**
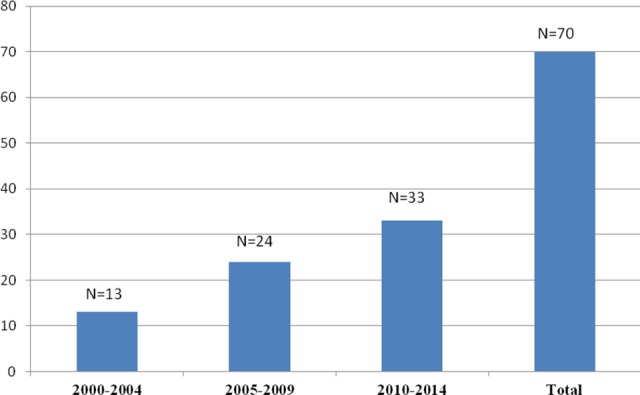
The number of patients receiving prophylaxis over the years.

Of the 481 patients, 367 (77.3%) were male and 114 (23.7%) were female. The median age of the patients at the time of diagnosis and at the time of the study was 4.1 years (range: 2 months to 20.4 years) and 13.4 years (range: 7 months to 31.3 years), respectively. The median duration of the follow-up time was 6.8 years (range: 2.5 months to 24.8 years). There were 134 (29.7%) patients with hemophilia A, 38 (8.5%) with hemophilia B, 57 (10.8%) with vWD, and 252 (51%) with RBDs. Distribution of the patients with RBDs was as follows: 185 (73.4%) FVII deficiency, 20 (7.9%) FX deficiency, 19 (7.5%) FXI deficiency, 14 (5.5%) fibrinogen deficiency, 6 (2.3%) FV deficiency, 4 (1.5%) FV and FVIII combined deficiency, 2 (0.7%) FXIII deficiency, and 2 (0.7%) FII deficiency. Factor levels were less than 1% in 128 patients, between 1% and 5% in 70 patients, and above 5% in 283 patients. Genetic analyses were performed in only 8 patients with FVII deficiency and 2 with hemophilia A. One hundred nineteen patients (47.2%) in the RBD group were asymptomatic at the time of diagnosis, 65 of whom were diagnosed before surgical procedure, 49 by family history, and 5 after prolonged bleeding during surgeries. Consanguinity rate for the RBDs was 47.2%. The prevalence rates of bleeding for patients with symptomatic RBD was as follow: epistaxis 63.9%, skin bleedings 48.8%, oral cavity bleeding 28.5%, CNS bleedings 16.5%, hematomas 15%, hemarthrosis 15%, GIS bleedings 4.5%, bleeding because of operations 3%, menorrhagia 2.2%, hematuria 2.2%, and iliopsoas bleedings 2.2%. Of the 133 patients with symptomatic RBD, 69 (51.8%) had grade II and 64 (48.2%) had grade III bleeding symptoms. Distribution of CFDs and general characteristics of patients are summarized in Tables 1 and 2, respectively. Of the 134 patients with hemophilia A, 7 (5.2%) patients developed inhibitor during the follow-up period. Immune tolerance induction (ITI) therapy was initiated in 4 patients and eradication of the inhibitor was achieved in 2 patients in the 6 months of ITI, 1 patient has still been on ITI therapy for 6 months, and 1 patient did not respond to ITI (Table 3). Patients who had undergone major or minor surgeries, radioisotope synovectomy, or circumcision are presented in Table 4. Primary prophylaxis was initiated in 10 patients with hemophilia A, whereas secondary prophylaxis was initiated in a total of 70 patients with a distribution of 48 with hemophilia A, 7 with hemophilia B, 2 with vWD, 5 with FVII deficiency, 4 with FX deficiency, 3 with fibrinogen deficiency, and 1 with FV deficiency (Table 5). In patients with RBD, 5 patients with FVII deficiency were given prophylaxis of recombinant-activated factor VII (rFVIIa) weekly or twice a week, 4 patients with FX deficiency was given prothrombin complex concentrate (PCC) twice a week, 3 patients with fibrinogen deficiency were given fibrinogen every 2 weeks, 1 patient with FV deficiency were given FFP weekly. Indications for prophylaxis were CNS bleeding in 8 patients, recurrent and various site of bleeding in 2 patients, recurrent hemarthrosis in 2 patients, and iliopsoas bleeding in 1 patient. Development of inhibitors and thrombotic incidents was not observed during bleedings and prophylaxy treatments in patients with RBDs. An allergic reaction to FFP developed in 1 FV deficiency case. The median duration of prophylaxis was 5 years (range: 2 months to 14 years). There were only 13 patients receiving prophylaxis between 2000 and 2004 years, which increased to 37 patients between 2005 and 2009 and reached a total of 70 patients with an increment of 150% between 2010 and 2014 compared to period between 2005 and 2009 (Figure 1).

**Table 1.** General Characteristics of Patients.

Median age at the time of study, years	13.4 (range: 7 months to 31.3 years)		
Median age at diagnosis, years	4.1 (range: 2 months to 20.4 years)		
Median follow-up time, years	6.8 (range: 2.5 months to 24.8 years)		
Sex, n (%)			
Male	367 (77.3)		
Female	114 (23.7)		
Factor activity, n (%)	Hemophilia A (n = 134)	Hemophilia B (n = 38)	Total (N = 172)
<1% of normal activity	99 (73.8)	20 (52.6)	119 (69.1)
1% to 5% of normal activity	19 (14.1)	9 (23.6)	28 (16.2)
>5% of normal activity	16 (11.9)	9 (23.6)	25 (14.5)
Clinical presentations of hemophilias and vWD (N = 229)	n (%)		
Symptomatic	213 (93)		
Asymptomatic	16 (7)		
Clinical presentations of rare factor deficiencies (n = 252)	n (%)		
Symptomatic	133 (52.8)		
Asymptomatic	119 (47.2)		
Inhibitor development^a^	7 (5.2)		

Abbreviation: vWD, von Willebrand disease.

^a^All patients were hemophilia A.

**Table 2.** General Characteristics of Patients With RBDs.

Characteristic	FI D*	FII D*	FV D*	FV + FVIII D*	FVII D*	FX D*	FXI D*	FXIII D*	Total
Patients (n)	14	2	6	4	185	20	19	2	252
Male	8	-	5	1	124	13	13	2	166
Female	6	2	1	3	61	7	6	-	86
Phenotype (n)									
Asymptomatic	4	1	2	2	94	4	12	-	119
Symptomatic	10	1	4	2	91	16	7	2	133
Bleeding severity^a^ (n = 133)									
Grade 1	-	-	-	-	-	-	-	-	-
Grade 2	6	1	2	1	47	5	7	-	69
Grade 3	4	-	2	1	44	11	-	2	64
F:C									
<5%	8	1	2	-	26	12	11	2	62
5% to 30%	4	-	4	4	38	7	1	-	58
>30%	2	1	-	-	121	1	7	-	132

Abbreviations: F, factor; F:C, factor level; D*, deficiency; RBDs, rare bleeding disorders.

^a^Bleeding severity of symptomatic patients.

**Table 3.** Characteristics of Patients With Inhibitor.

Case	Age, years	F:C	Duration of Inhibitor, years	Genetic	Peak Inhibitor (BU)	Prophylaxis (aPCC)	ITI Therapy
1	8	0.1%	4	-	60	+	−
2	11	0.1%	6	-	80	+	+
3	9	0.1%	3.5	-	32	+	+
4	20	0	16	Large deletion	60	+	+
5	3	0.3%	1	-	98	+	−
6	26	0	11	Large deletion	176	+	−
7	13	0.5%	3.5	-	6	+	+

Abbreviations: aPCC, activated prothrombin complex concentrate; BU, Bethesda unit; F:C, factor level; ITI, immune tolerance induction.

**Table 4.** Distribution of Surgeries and Nuclear Medicine Procedures.

	Hemophilias	RBDs	Total
Major surgery	31	30	61
Minor surgery	23	34	57
Circumcision	99	59	158
Radioisotope synovectomy	55	1	56

Abbreviation: RBDs, rare bleeding disorders.

**Table 5.** Distribution of Patients Receiving Prophylaxis.

	Primary Prophylaxis, n (%)	Secondary Prophylaxis, n (%)
Hemophilias and vWD (n = 229)		
Hemophilia A	10 (4.3)	48 (20.9)
Hemophilia B	–	7 (3)
vWD	–	2 (0.8)
Total	10 (4.3)	57 (24.8)
Rare bleeding disorders (n = 252)		
Fibrinogen deficiency	–	3 (1.1)
FV deficiency	–	1 (0.3)
FVII deficiency	–	5 (1.9)
FX deficiency	–	4 (1.5)
Total	0 (0)	13 (5.1)

Abbreviations: F, factor; vWD, von Willebrand disease.

## Discussion

Congenital factor deficiencies remain a popular area of research due to widespread increases in prophylactic treatments as well as ongoing studies on new drugs, long-acting FVIII and FIX concentrates, and gene therapy. In the 2014 World Federation of Hemophilia (WFH) annual report, 4860 patients with hemophilia A, 878 with hemophilia B, 1119 with vWD, 850 with FVII deficiency, 164 with FX deficiency, 180 with FXIII deficiency, 51 with FXI deficiency, 29 with FV deficiency, and 1 case of FV + FVIII deficiency were reported from Turkey.^24^ The rate of patients with vWD among CFDs was lower both in our national data and in the current study compared to patients with hemophilia A, which was considered to be due to diagnostic difficulties, including laboratory inadequacy, few number of reference hematology laboratories, and undiagnosed patients with vWD who do not require admission to hospital because of mild bleeding symptoms. Further, these numbers included cases that were recorded in the registry of Turkish Ministry of Health and did not reflect heterozygous cases and cases with borderline factor levels. The data from our clinic support this opinion. We examined the distribution of cases that were followed up since 1990 and many of them were RBD cases, especially FVII deficiency cases. Along with our hospital that serves a maternity hospital, our active pediatric surgery clinic has led to the early diagnosis of many RBD cases. Sixty-five of our asymptomatic RBD cases were diagnosed before surgical procedures, while 49 were diagnosed by family history and 5 during surgical procedures. One hundred eighteen patients underwent major or minor surgical interventions and 158 had been circumcised. Fifty-six radioisotope synovectomies were performed in 36 patients after evaluation by our hospital’s hemophilia committee.

The first prophylactic treatment that was administered to patients with severe hemophilia in Sweden in the 1960s has become the standard for treatment.^25^ The current discussion for patients with hemophilia A and B revolves around ways of making prophylactic treatment more effective and improving the continuity of treatment compliance.^26–28^ In accordance with the tendency around the world, prophylactic treatments have gained momentum in our country as well. The coverage of prophylactic treatment by national health insurance and the efforts of patients’ associations have contributed to this development. The prophylaxis recommendations of Turkish Society of Pediatric Hematology have been guiding prophylactic treatments in children. According to the WFH 2013 annual report, the percentage of patients on prophylaxis was 80% among those under 18 years old and was 75% among those over 18 years old in our country.^29^ In the 2013 and 2014 WFH annual reports, per capita FVIII consumption in Turkey was 3.793 and 3.123, respectively.^24,30^ According to the latter 2 reports, although our country has been in the upper middle income economy category, mean per capita FVIII use in Turkey was twice more compared to the countries listed in the same economic category. In the 2010 report about the “optimal use of blood and blood products,” the special recommendation of minimum acceptable per capita consumption of FVIII in hemophilia was 2 units.^30^ Based on this recommendation and WFH reports, we believe that our country has made great progress in the treatment of hemophilia. The results of the survey completed by 35 European countries in 2013 also determine the stage of these countries in hemophilia treatment.^10^ Upon examination of our country’s results, we see that the applicability of home treatment was 51% to 75%, the accessibility of prophylaxis was 51% to 75% in children and was 1% to 25% in adults, and the accessibility of immune tolerance induction therapy (ITT) administration was 1% to 25%. While the medical specialist support required for hemophilia treatment has been deemed generally adequate, it has been observed that most of the factors used in the treatment are accessible in our country. Sixty-three patients in our hemophilia group (30.6%) and 13 patients in our RBDs group (5.2%) have received prophylactic treatments. The need of prophylaxis in patients with RBDs is emphasized occasionally. Publications about the prophylaxis were as case reports exception of FXIII and FVII deficiency.^31,32^ The information we have about prophylaxy is either limited, short termed, or only covers the period of a surgical intervention. We believe that prophylaxy is an option that should be evaluated in patients who present with severe bleeding. In our series, 5 patients with FVII deficiency were given prophylaxis of rFVIIa weekly or twice a week, 4 patients with FX deficiency was given PCC twice a week, 3 patients with fibrinogen deficiency were given fibrinogen every 2 weeks, and 1 patient with FV deficiency was given FFP weekly. There are no determined standard prophylaxy programs for patients with RBDs. It is recommended in life-threatening bleedings or those that could result in disabilities. Prophylactic experiences particularly for FVII, FX, and FV deficiency should be evaluated on a patient-by-patient basis.

In the present study’s patients with hemophilia, the rate of inhibitor development was 5.2% and all were patients with hemophilia A. In the 2 national surveys that were conducted in 2003 and 2010 in Turkey, the rate of inhibitor development was 10.3% and 14%, respectively.^33,34^ Taken together, inhibitor development rate both in the current study and in the national surveys conducted in our country was lower, when compared to the rates of 25% to 33% in patients with severe hemophilia A reported by the current literature.^35–37^ The explanations for the low rate of inhibitor development were lack of access to clotting factor concentrates and laboratory inadequacies. Additionally, we think that patients with transient inhibitors and low responding inhibitors are missed due to low frequency of inhibitor testing. The ITI therapy could only be administered to 4 of our 7 patients with inhibitors. The present national health insurance in our country does not state clear rules for the coverage of ITI treatments, and there are problems with the patients as well. This was not the case for the RBDs group, as the number of patients in need of prophylaxis was relatively low. For some factor deficiencies, the lack of specific concentrates and the unwillingness of some patients to continue treatment restrict the prophylactic treatment rate in the RBD group.^23^ Our clinic began administering prophylactic treatments with 1 patient having hemophilia A in 2001. The number of patients who were started on prophylactic treatments between 2010 and 2014 was observed to be as much as the sum of those who were started before 2010 (37 vs 33). The dramatic increase in the administration of prophylactic treatment over the years shows that our clinic has been able to implement up-to-date treatment approaches. Our biggest shortcoming in terms of CFDs has been that we cannot determine the mutation types of the patients. The importance of genetic studies for the awareness of the distribution in our country should not be forgotten.^38^

There are some limitations in the current study. Laboratory inadequacies including measurement of coagulation factor activity and inhibitor testing were the most essential issues. There are few reference laboratories available in our country for the measurement of coagulation factor activity and inhibitor testing, and getting laboratory test results from those centers usually take a long time, which leads to delayed diagnosis and causes patient anxiety and noncompliance, particularly for the patients with borderline vWD deficiency and RBDs, who need more diagnostic tests. These factors might have effects on the current study’s results. Further, genetic mutation testing could be performed in very few patients and only in special situations, leading to difficulty in proper genetic counselling to families.

In conclusion, the applicability of up-to-date treatments is highly important for the patients with CFDs to lead a healthy and normal life. Thankfully, with the increased availability of safe clotting factor concentrates and the switch of treatment from on demand to prophylaxis over the years, majority of these patients, particularly hemophiliacs, are now able to live far from bleedings and related risk, especially in developed countries. Inhibitor development to the replaced clotting factor, occurring in up to one-third of patients with severe hemophilia A, remains the most significant complication of hemophilia treatment. New long-acting agents and gene therapy for hemophilias seems to be the promising candidates in the future treatment approaches, which can provide a normal quality of life. The rarity and variability in clinical presentations in patients with RBDs are significant issues, making the diagnosis and management of these patients difficult. In this context, multinational collaborative studies are needed for these patients to get the same success achieved for the hemophiliacs.
